# Associations between Cultural Identity, Household Membership and Diet Quality among Native Hawaiian, Pacific Islander, and Filipino Infants in Hawaiʻi

**DOI:** 10.3390/children9010048

**Published:** 2022-01-02

**Authors:** Jessie Kai, John J. Chen, Kathryn L. Braun, Joseph Keaweʻaimoku Kaholokula, Rachel Novotny, Carol J. Boushey, Marie K. Fialkowski

**Affiliations:** 1 Department of Human Nutrition, Food and Animal Sciences, College of Tropical Agriculture and Human Resources, University of Hawaiʻi at Mānoa, 1955 East West Road, AgSci 216, Honolulu, HI 96822, USA; jessiek@hawaii.edu (J.K.); novotny@hawaii.edu (R.N.); 2 Department of Quantitative Health Sciences, John A. Burns School of Medicine, University of Hawaiʻi at Mānoa, 651 Ilalo Street, MEB Suite 411, Honolulu, HI 96813, USA; jjchen@hawaii.edu; 3 Office of Public Health Studies, Thompson School of Social Work & Public Health, University of Hawaiʻi at Mānoa, 1960 East-West Road, Honolulu, HI 96822, USA; kbraun@hawaii.edu; 4 Department of Native Hawaiian Health, John A. Burns School of Medicine, University of Hawaiʻi at Mānoa, 677 Ala Moana Blvd., 1016, Honolulu, HI 96813, USA; kaholoku@hawaii.edu; 5 Nutrition Support Shared Resource, University of Hawaiʻi Cancer Center, 701 Ilalo Street, Honolulu, HI 96813, USA; Cjboushey@cc.hawaii.edu

**Keywords:** complemenary feeding practices, dietary diversity, infants

## Abstract

Public health efforts to reduce diet-related health disparities experienced by indigenous peoples could be enhanced by efforts to improve complementary infant feeding practices. The latter is possible through interventions informed by cultural determinants. This cross-sectional secondary analysis explored possible determinants of the complementary feeding practices of Native Hawaiian, Pacific Islander, and/or Filipino infants (NHPIF) in Hawaiʻi, ages 3–12 months. The objective was to determine the association between caregiver cultural identity and infant household membership with indicators of infant diet healthfulness. The cultural identities, infant household memberships, early infant feeding practices and additional demographic information (infant age and sex, household income) were assessed via an online questionnaire. Surrogate reporting of the infants’ diets over four days was evaluated using an image-based mobile food record (mFR). Data collected by the mFR were evaluated to derive the World Health Organization’s minimum dietary diversity (MDD) indicator and food group consumption. Data were summarized by descriptive statistics and analyzed using multivariate linear and logistic regressions. Seventy infant participants, ages 3–12 months, and their primary caregivers completed the study. Of these, there were 56 infant participants between the age of 6–12 months. Approximately 10% of infants, ages 6–12 months, met MDD for all four days. Meeting MDD and the number of food groups consumed were significantly associated with age. Caregiver cultural identity, infant household membership and infant sex had non-significant associations with indicators of infant diet quality. Findings inform the influences shaping dietary patterns of Native Hawaiian, Pacific Islander and Filipino infants in Hawaiʻi.

## 1. Introduction

Unhealthy long-term eating patterns, resulting in a multitude of negative consequences and sequelae, often originate during childhood with unhealthy feeding practices [1,2]. Important characteristics of such infant feeding practices include the timing and type of complementary food introduction, both of which impact the foundations of nutritional status and health [3]. Complementary feeding is the stage in infant feeding where infants are transitioning from consuming only human milk and/or infant formula to consuming other nutritive foods and beverages in addition [3,4].

Diet quality is crucial in relating complementary infant feeding practices to childhood health and nutritional status. The quality of the diet and the quantity of foods consumed are nutritional contributors to diet-related illnesses, such as obesity and diabetes [5]. Diet quality contributing to the risk of becoming obese among older individuals can be seen in the associations between low intakes of nutrient-dense foods, high intakes of energy-dense foods, and indicators of body size [6]. Among infants, dietary diversity is an indicator of the quality of the diet. Diet diversity is a proxy measure of the nutrient density of the foods and liquids consumed. A diverse diet is associated with a better quality diet and can be used to predict the micronutrient density among infants [4]. However, the diversity of infant diets in the first year of life was observed to be low with sugar-sweetened beverage commonly consumed as the first food, and low consumption of fruits and vegetables [7].

Sound nutritional practices during the first years of life are paramount for healthy growth and development. Research has shown that establishing healthy infant and young child feeding/eating practices during the first 1000 days of life leads to healthy outcomes experienced throughout an individual’s life [6]. In a similar but opposing manner, unhealthy feeding/eating practices established during infancy and childhood may lead to adverse health outcomes [8]. Thus, nutrition early in the lifespan sets the health trajectory of an individual.

The influence nutrition has on the health of infants can be seen by the effect nutrient consumption has on energy availability, metabolism, and growth measures. Rapid growth of Native Hawaiian, Pacific Islander and/or Filipino infants, 12–23 months old, was predictive of obesity at 4–5 years old [2]. An estimated 14% of children, 2–8 years old, in the US-Affiliated Pacific region were observed to be obese, with the prevalence of obesity higher among the older children in the study [9]. Together, these findings are concerning given the progression of adverse health conditions (i.e., obesity, hypertension, type 2 diabetes) experienced by children and their persistence into adulthood [10]. The prevalence of obesity during adulthood and type 2 diabetes in the Pacific region is among the highest in the world [11]. Native Hawaiian and Pacific Islanders were found to be 3.32 times more likely to die from diabetes compared to the general population in Hawaiʻi [12]. Rapid infant growth is believed to be a precursor to childhood obesity, and obesity during childhood likely continues into adulthood [13]. A previous study on complementary infant feeding in Hawaiʻi observed race/ethnic differences between infants identified as White, Japanese, Filipino and Native Hawaiian, with the latter race introducing solids earlier than 4 months of age [14]. 

A key contributor to an obesogenic environment is the shift from traditional diets high in fiber to diets high in sugars, fat, and animal food sources seen in Western-style eating patterns. These dietary changes result from economic and infrastructure changes and urbanization, ushering in changes to the local food ecosystem [15]. The shift away from traditional foods is impactful, as these foods support spiritual connections between the people preparing and consuming the foods, the natural source of the foods, and the culture and traditions of the people [16]. However, a dilemma exists among indigenous peoples’ food and their wider environment, as Western-based food types and eating habits have undermined traditional hunting, gathering and growing systems long practiced by these racial and ethnic groups [16]. The normalization of non-traditional diets among indigenous people, due to the colonization of indigenous lands and food systems, has ushered in the transition away from cultural dietary patterns to those of another dominating culture [16,17,18,19].

Possible determinants of eating behaviors include social environments such as household environments. From 2009 to 2011, the US Census Bureau found that 8% of households with a multiracial householder were multigenerational [20]. Among Native Hawaiian and other Pacific Islander households, about 18% consisted of three or more generations, while 3.7% of non-Hispanic White households and 9% of Black and Asian households were multigenerational [20,21]. Pacific Islanders have reported family, particularly female family members, as central in providing support and advice, especially in regard to infant care and feeding [22]. 

According to a Consumer Expenditure Survey, such multigenerational households spend less on childcare [23]. Grandparents were found to be an adaptive strategy for low-income or single-parent households in offsetting living costs, allowing greater financial investments in the children [24]. The practice and perpetuation of culture, such as preparing and consuming cultural foods, may be supported in a living arrangement where multiple persons have knowledge of and practice cultural traditions and customs.

It is unclear how these influences shape nutrition among Native Hawaiian, Pacific Islander and Filipino infants (NHPIF). This examination is a beginning step in establishing good feeding/eating practices, and progress towards preventing unhealthy behaviors. The researchers here address the need for literature on social and cultural environments of infants to identify determinants of dietary behaviors. The study, which is a secondary analysis, aimed to examine the association between the cultural identity/identities of caregivers, the membership of households, and dietary diversity among NHPIF infants in Hawaiʻi. Currently, this is the first study to focus on multigenerational households, caregiver cultural identity and the association of these with infant complementary feeding practices in Hawaiʻi. 

## 2. Materials and Methods

### 2.1. Study Design and Setting 

Institutional Review Board (IRB) approval from the University of Hawaiʻi was received prior to the collection of data (IRB reference number: 2017-00845). The criteria for participation included that the infant participant resided on the island of Oʻahu at the time of data collection, the caregiver of the infant participant had to be 18 years of age or older, have an iOS mobile device, and have reliable access to the internet and to the iOS device. The infant participant had to start complementary feeding prior to enrollment and be reported by the caregiver as at least part Native Hawaiian, Pacific Islander or Filipino.

### 2.2. Recruitment and Consent 

A convenience sample of NHPIF infants was recruited through community-based events (e.g., Baby Expo), programs (e.g., Women, Infants, and Children), professional networks (e.g., colleagues), and personal networks (e.g., friends and family). Consent was obtained in writing from the caregivers for both their participation and their infant’s participation prior to collecting any data. Data were collected between March 2018 and February 2019.

### 2.3. Study Outcomes 

Caregivers completed online questionnaires using a secure online web application. Questions included feeding behaviors prior to enrollment in the study, place of birth/delivery, annual household income, and participation in food assistance programs. Caregivers were asked who currently lives in the child’s home and how they are related to the child. Caregivers were also asked to select what specific race/ethnic group(s) best describes their child. The cultural identity scales used here were validated in studies examining the degree of Native Hawaiian cultural and US, Mainland mainstream cultural identifications [25,26]. Briefly, the 8-item cultural identity questionnaire had two subscales: a 4-item ethnic cultural identity subscale and a 4-item US cultural identity subscale. Each subscale asked the caregivers about their degree of identity with involvement in, feelings toward, and knowledge about each cultural group, and the impact each cultural group has on their lifestyle. Responses to each item were reversed scored, so that 1 corresponded to very knowledgeable, very positive, or very involved and 5 corresponded to not knowledgeable at all, very negative, or disinterested. The total possible scores ranged from 5 to 25, with lower scores indicating a stronger identity [25,26]. 

Household composition was examined by categorizing the household into two groups. Modifying Lane and colleagues’ [27] household composition categories, the following categories were applied in this study: households with only the parent(s) and parent(s) and sibling(s) and households with extended family members including maternal and paternal grandparents, aunts, uncles, cousins, and nonrelative individuals. This variable was used as a proxy for the number of individuals likely to be physically interacting with, caring for, and sharing a common environment with the infant participants on a regular basis. 

Infant dietary assessment was completed through surrogate (i.e., caregiver) reporting with the mobile food record (mFR). The mFR is an application designed specifically for the assessment of dietary intake from the Technology Assisted Dietary Assessment (TADA) project (http://tadaproject.org/; accessed on 29 December 2021), which uses the camera on a mobile device to capture food and beverage intake, which is then used to estimate energy, nutrients, food and beverage intakes [28,29]. The mFR was loaded onto the caregiver’s iOS mobile device, and training on the mFR application was completed prior to data collection. 

Caregivers were instructed to take before and after images of all foods and beverages the participant consumed over a 4-day collection period (Thursday–Sunday). After the collection period concluded, a member of the research team reviewed the images with caregivers to verify content, as needed, and to probe for any forgotten foods or beverages. At the end of the data collection period, caregivers were compensated with a $40 gift card.

### 2.4. Analysis 

This study used the global metric Minimum Dietary Diversity (MDD) score from the World Health Organization (WHO) indicators for assessing infant and young child feeding practices [30]. The MDD score provides an indication of the infant’s diet quality based on the awareness that consuming a wide range of foods is a tenet of a healthy diet, as it increases the likelihood of meeting nutrient needs. For infants, the number of food groups consumed can predict the nutrient density of the diet. 

The MDD indicator is appropriate for use with infants and young children between the ages of 6–23 months old. The revised MDD indicator has 8 defined food groups: (1) grains, roots, and tubers; (2) legumes and nuts; (3) dairy products (milk, including formula, yogurt, cheese); (4) flesh foods (meat, fish, poultry, liver/organ meats); (5) eggs; (6) vitamin A-rich fruits and vegetables; (7) other fruits and vegetables; and (8) human milk [30]. All solid foods and liquids consumed over the four days of data collection were enumerated using the mFR. Human milk was counted as consumed daily across the four data collection days for those participants reported as being breastfed. The consumption of human milk was extrapolated from the infant feeding behavior survey question “Is your child still breastfeeding?” The WHO MDD guidelines specifically state that the indicator was met if five or more food groups were consumed, on average, each day by infants 6–12 months old [30]. For all the infant participants, ages 3–12 months old, the average number of food groups consumed over the data collection period was analyzed. 

Descriptive statistics were used to summarize the frequencies, means, and standard deviations (*n* = 70). The response variables were the infants’ number of daily food group consumption, a continuous variable, for infants 3–12 months old, and the MDD indicator, a dichotomous variable, for infants 6–12 months old. The explanatory variables were the continuous cultural identity scores of the caregivers, and the infants’ household composition, a categorical variable. Multivariable linear regression was used to determine if an association exists between the explanatory variables and the number of food groups consumed, after adjusting for sex and age. Logistic regression was conducted to assess the association between the explanatory variables and meeting or not meeting the MDD indicator, after adjusting for sex and age. Statistical significance was set at *p*-value < 0.05. All analyses were conducted in IBM SPSS Statistics Version 27.0 (SPSS Inc: Chicago, IL, USA).

## 3. Results

### 3.1. Descriptive Statistics

Seventy infant participants completed the study. Of those who consented, 13 were lost to follow-up, resulting in an attrition rate of about 16%. The majority of the infant participants were between 6 and 12 months old and about half were males (Table 1). More than 70% of infant participants were identified by their caregivers as Part-Native Hawaiian or Native Hawaiian, and about 50% were reported as part-Filipino or Filipino. English was the most commonly spoken language in the participants’ homes. 

More than 60% of the infants were fed both human milk and infant formula. About 30% of the participants were fed only human milk. Half of the participants were still consuming human milk during the study. About half of the infants were fed non-human milk or infant formula foods (i.e., rice cereal, poi, baby food purees) before turning 6 months old. Across all the participants and the different modes of feeding (i.e., human milk, infant formula or both), the mean age when non-human milk or infant formula foods were introduced was similar at about 5 months of age (Table 1). Household membership was categorized into two groups (Table 1). More than half of the infant participants lived in a household consisting of parent(s) or parent(s) and sibling(s). 

### 3.2. Dietary Healthfulness 

Approximately four food groups on average were consumed across the four days of data collection (Table 2). The mean number of days the MDD indicator was met by participants, ages 6–12 months, was 2.0 (SD = 1.6). About 10% of participants met the MDD all four days of the data collection (Table 3). No difference was observed in the proportion meeting the indicator by the day of the week (Table 3).

### 3.3. Multivariate Regression Models 

Consumption of all eight food groups was not observed among the participants. In the multivariate linear regression models, older age was significantly associated with more food groups consumed (Table 4). In the multivariable logistic regression models, only age was significantly associated with the likelihood of meeting the MDD indicator (Table 5).

## 4. Discussion

This is the first study examining the relationship between cultural identity and household composition on diet quality of NHPIF infants in Hawaiʻi. In this examination of potential determinates of dietary diversity of infants, age was significantly associated with diet outcomes. This is expected as the infant gains motor control and develops advanced eating skills (i.e., mastication, swallow, acceptance of broader food consistencies, texture, taste). Other studies have made similar observations [31,32,33]. No associations were observed between household composition, degree of ethnic identity and US, Mainland identity, and diet quality among the 70 infant participants. 

This study examined indicators of acculturation (e.g., multigenerational households and cultural identity) to investigate cultural influences on diet. The cultural identity scales used here were adapted from the studies by Kaholokula and colleagues [25,26]. Kaholokula’s scales are used as surrogate factors to measure the degree of ethnic cultural identity and US cultural identity of Native Hawaiians. These scales use a bidirectional approach as people can simultaneously identify with two identities to varying degrees [25,26]. Although there are other scales to examine indicators of acculturation, the scales used in this study are more representative of the history and experiences of the populations of focus. 

The lack of literature on indicators of acculturation and diet quality among infants persists. Among the few studies published, Zhang and Benton observed feeding styles and diet quality were not correlated with acculturation among first-generation Chinese immigrant mothers in England [34]. Zhang and Benton assessed acculturation using the Mutual Intercultural Relations in Plural Societies (MIRIPS) Questionnaire. Although Zhang and Benton categorized their participants by acculturation modes, and this study did not, similar findings were observed. However, among US Latino caregivers, low acculturation scores were associated with a parent feeding style related to poorer infant diet quality [35]. Possibly the different acculturation tools used could explain the different associations [34,35]. The Short Acculturation Scale for Hispanics (SASH) is based on language preference, which introduces variability of the measurement and proves difficult to make conclusions and comparisons [35]. The literature highlights other factors potentially affecting dietary choices including food availability and familiarity, marketing and advertising, and comprehension of nutrition education [36,37]. Perhaps the ethnic identity a caregiver chooses to associate with is not the strongest influence on their infant’s dietary quality. 

Although this study observed no association between household membership and diet quality, other studies have found otherwise. Lane and colleagues observed differences in the fecal bacterial composition of infants of different household compositions [27]. Research on Aboriginal infants and children in northern Australia found that those living in a household of 3–5 people had higher meal frequencies compared to those in larger households [38]. However, unlike the research here and by Lane, the relationships between the individuals and the infants and children were not defined [38]. A possible explanation is that larger households with more individuals to feed present additional economic challenges, resulting in less quality foods consumed. Securing traditional and cultural foods for some ethnic groups is financially costly and time-consuming. 

The joining of family and non-family members into a single cohesive unit potentially misrepresents the living situation [39]. Family encompasses relationships across generations [40]. These intergenerational relationships are diverse in structure and functions, with certain relationships being valuable resources for families [40]. One such valuable intergenerational relationship is that with grandparents. Grandparents may potentially have an important part in family life by providing financial, emotional and practical care and support to their adult children and grandchildren [41]. The benefits of multigenerational households include increased spending on education, decreased spending on childcare, companionship, support and increased likelihood of daily conversations supporting transgenerational exchanges of knowledge and perspectives [21,23,40,41]. Older generations potentially shape infant feeding practices (i.e., timing of solid food introductions). This is seen in the important role female family members have in infant care in Pacific Islander families [22]. 

Although there are clear advantages for multigenerational family housing where the grandparents provide support rather than are in need of support, these types of living arrangements are associated with socioeconomic disadvantage and lack of personal privacy and control [39,41]. Research focused on African-American families observed that non-maternal caregivers such as grandmothers, fathers and licensed childcare providers had a negative effect on age-appropriate infant feeding practices [42,43]. This highlights the dynamic nature of family, and how individuals with different roles and varying degrees of control interact with one another to impact the growth development of infants. 

At the time of this research and dietary analysis, the Dietary Guidelines for Americans 2020–2025 were not designed, and so the researchers used the WHO dietary diversity indicator as the measurement of diet healthfulness [4,30]. The underlying assumption of the WHO guidelines is that infants will be exclusively fed human milk and/or infant formula until the age of 6 months. Furthermore, WHO designed the dietary diversity score and other measures of Infant and Young Children Feeding Practices (IYCFP) for low-income countries, making it inherently more sensitive in settings where food availability is limited [4]. The MDD indicator is designed for infants 6–23 months old [30]. Participants in this study included infants 3–5 months old, and therefore, the dietary diversity indicator could not be applied to these participants. The age restriction limits the investigating into the influences shaping early feeding practices in this sub-population known to introduce solid foods at an early age [14,31]. Exclusively human milk and/or infant formula feeding from birth to 6 months was not seen among the study’s participants, with 50% of the infants consuming non-human milk and/or infant formula foods before 6 months (i.e., did not meet the WHO timing recommendation for complimentary feeding). This highlights an issue with the indicator as non-human milk and/or infant formula food introductions before the age of 6 months may result in higher dietary diversity scores at the 6 months timepoint. Thus, if an infant started eating non-human milk and/or infant formula at 3 months old, by the time the infant is 6 months, s/he will have 3 months of non-human milk and/or infant formula food exposure compared to an infant starting at the recommended age of 6 months. Therefore, higher dietary diversity scores could be associated with early feeding practices, thus potentially misleading the association between early feeding practices and more diverse diets. 

As mentioned previously, the consumption of human milk was constructed from the online survey in which caregivers answered if they were currently breastfeeding. The use of the term breastfeeding presents a limitation and the researchers here recognize that the provision of human milk occurs through multiple means (e.g., breastfeeding, chest feeding, and/or bottle feeding). Using more inclusive terms may have captured infants fed human milk via other methods. 

This study was limited by the small sample size of 70 participants between the ages of 3–12 months old, and a sub-sample of 56 between the ages of 6–12 months old. The participants and caregivers in this study were not equally represented in their demographics (i.e., age, sex, education, occupation, marital status, racial/ethnic identification(s), household membership, income). The researchers acknowledge the potential influence of these caregiver variables (sex, age, education, occupation, etc.) and/or family factors (income, family size, etc.) as limitations of this study. 

The convenience sample used in this study may not be representative of the targeted racial or ethnic groups. This study focused on NHPIF infants as a group and was not designed for comparisons between different ethnic groups. Race/ethnicity was self-reported, with over 25% of participants being from more than one racial group. As a result, the infants’ race(s) were not included into the analysis. The researchers recognize that there could be selection biases or confounding. Future studies should consider using disaggregated race/ethnicity data with purposeful sampling. Furthermore, the sampling has inherent limitations in the statistical analysis approach, including the regression approach possibly being influenced by outliers. Researchers recognize that the model described an association with little statistical power to demonstrate statistically significant relationship(s). The associational effect observed may be influenced by section biases and variables having predictor and confounder influences. Confounding variables unknown to the researchers were also not adjusted for in the model. 

This study is a secondary analysis of a cross-sectional pilot study focused on infants of racial and ethnic groups, a poorly represented demographic in the current public health literature. The work here not only adds a unique focus on health issues of this population but also examines unique characteristics of this population and the potential of these characteristics to modify the caregiver and infant feeding relationship. This study adds to the literature examining the relationships between adverse health outcomes (e.g., diet-related illnesses) and aspects of life influenced by culture (e.g., food intake, physical activity, language comprehension, education, knowledge) [44,45]. People change their health behaviors including dietary habits and physical activity to reflect the dominant culture of their surrounding environment [14,42,43].

Finally, this study used an innovative mobile technology-based dietary assessment tool (e.g., mFR) to improve the accuracy of dietary intakes and reduce the risk of recall bias [31]. The dietary data collected was the first time an image-based dietary assessment tool was used to enumerate the intake of infants. The mFR used in this study was designed for iOS mobile devices, limiting the study to those with this mobile operating system. Future studies using the mFR for infant dietary intakes should include users of other mobile operating systems. Although the explanatory variables of interest were not significantly associated with diet diversity, recognizing the transition individuals from racial and ethnic groups undergo as their environments and the surrounding cultures change is crucial to understanding how people adapt to the dominant culture(s) around them.

## 5. Conclusions

The influence of culture on health and lifestyle behaviors emphasizes the importance of understanding the attitude and beliefs surrounding a person’s cultural identify. Primary health prevention strategies stand to benefit from understanding the influence of people’s social and cultural environments on their dietary behaviors, and from respectfully incorporating culture, traditions and ways of living in the design and administration of such strategies. These strategies should encompass complementary infant feeding practices, which are important milestones in the development of an individual’s health and thus impact long-term health and nutrition status. 

## Figures and Tables

**Table 1:** Demographics and feeding practices of infants 3–12 months of age (*n* = 70).

Characteristic	*n*(%) ^a^	Mean (SD)
Age (months)		7.4 (2.1)
Age Group: 3–5 Months	14 (20.0)	
Age Group: 6–12 Months	56 (80.0)	
Sex		
Male	38 (54.3)	
Female	32 (45.7)	
Race/Ethnicity ^b^		
Part-Native Hawaiian or Native Hawaiian	50 (71.4)	
Pacific Islander Only ^c^	4 (5.7)	
Part-Filipino or Filipino	35 (50.0)	
Primary Language Spoken in Home		
English	64 (91.4)	
Non-English	3 (4.3)	
Missing Response	3 (4.3)	
Human Milk or Formula Feeding		
Human Milk Only	22 (31.4)	
Human Milk and Formula ^d^	44 (62.9)	
Formula Only	4 (5.7)	
Currently Receiving Human Milk	40 (57.1)	
Timing of Complementary Food Introduction		
Before 6 Months	37 (52.9)	
3 Months or Less	4 (5.7)	
4–5 Months	33 (47.1)	
6+ Months	30 (42.9)	
Missing Response	3 (4.3)	
Timing of Complementary Foods (in Months) by Milk Type		
Human Milk Only (*n* = 21, Missing Response = 1)	4.9 (1.4)	
Human Milk and Formula (*n* = 42, Missing Response = 2)	5.2 (1.2)	
Formula Only (*n* = 4)	4.6 (1.3)	
Received Assistance to Pay for Food ^e^	26 (37.1)	
Household Membership Category		
Parent(s) only or Parent(s) and Sibling(s) ^f^	44 (62.9)	
Extended family ^g^ included	26 (37.1)	
Ethnic Group Cultural Identity		
Score		7.8 (2.5)
US, Mainland Cultural Identity		
Score		10 (3.2)

^a^ May not add up to 100% due to rounding. ^b^ More than one race/ethnicity may have been self-selected; therefore, will not add up to 100%. ^c^ Participants only self-reported identifying with Pacific Islander ethnic groups, including Chamorro, Samoan, Tongan, Maori, Tahitian, and others not specified. ^d^ Includes infants who have received infant formula at some point or who are currently receiving infant formula. ^e^ Includes assistance from the Supplemental Nutrition Education Program (SNAP) and the Special Supplemental Nutrition Program for Women, Infants, and Children (WIC). ^f^ Includes households with only the parent(s) and households with parent(s) and sibling(s). ^g^ Includes households with maternal and paternal grandparents, aunts, uncles, cousins, and nonrelative individuals. SD = Standard Deviation.

**Table 2:** Number of food groups recorded for infants (*n* = 70) across the 4 days and by day of the week.

Consumption of Food Groups	Mean (SD)
Across all 4 days	3.7 (1.3)
By day of week	
Thursday	3.9 (1.4)
Friday	3.8 (1.3)
Saturday	3.6 (1.2)
Sunday	3.6 (1.2)

**Table 3:** Frequency of number of days infants, ages 6–12 months (*n* = 56), met the Minimum Diet Diversity (MDD) ^a^ and by day of week.

Meeting MDD Indicator	*n* (% ^b^)
Days Met	
0	22 (39.3)
1	12 (21.4)
2	8 (14.3)
3	8 (14.3)
4	6 (10.7)
Met MDD By Day of Week	
Thursday	24 (10.7)
Friday	19 (8.5)
Saturday	18 (8.0)
Sunday	15 (6.7)

^a^ Meeting MDD if the infant is reported to have consumed five or more of the eight food groups. ^b^ Numbers may not add up to 100% due to rounding.

**Table 4:** Multivariable linear regression examining the association between the number of food groups consumed and selected explanatory variables ^a^ in infants 3–12 Months (*n* = 70).

	Model 1 B (SE) 95% CI		Model 2 B (SE) 95% CI		Model 3 B (SE) 95% CI		Model 4 B (SE) 95% CI		Model 5 B (SE) 95% CI	
Constant	2.0 (0.4)	1.2–2.8	1.6 (0.5)	0.7–2.6	1.3 (0.5)	0.2–2.3	1.4 (0.7)	0.4–3.0	1.3 (0.7)	−0.2–2.7
Age	0.2 (0.05) ***	0.1–0.3	0.2 (0.05) ***	0.1–0.3	0.2 (0.06) ***	0.1–0.4	0.2 (0.06) ***	0.1–0.4	0.2 (0.06) ***	0.1–0.4
Sex			0.3 (0.2)	−0.2–0.7	0.3 (0.2)	−0.2–0.7	0.3 (0.2)	−0.2–0.8	0.3 (0.2)	−0.2–0.8
Household Membership					−0.03 (0.2)	−0.5–0.5	−0.03 (0.2)	−0.2–0.4	−0.05 (0.3)	−0.5–0.5
Ethnic Cultural Identity							−0.2 (0.05)	−0.1–0.1	−0.03 (0.05)	−0.1–0.1
US, Mainland Cultural Identity									0.02 (0.04)	−0.1–0.1
R-Squared	0.24		0.26		0.27		0.25		0.25	
Adjusted R-Squared	0.23		0.24		0.23		0.21		0.20	

^a^ Explanatory variables are age, sex, household membership categories, ethnic cultural identity categories and US, Mainland cultural identity categories of caregivers. *** indicates *p*-value < 0.05. Model 1: Age. Model 2: Age + Sex. Model 3: Age + Sex + household membership categories. Model 4: Age + Sex + household membership categories + ethnic cultural identity categories. Model 5: Age + Sex + household membership categories + ethnic cultural identity categories + US, Mainland cultural identity categories.

**Table 5:** Multivariable logistic regression results examining the association between meeting the MDD indicator and selected explanatory variables ^a^ in infants 6–12 Months (*n* = 56).

	Crude Analysis		Adjusted Analysis	
	Odds Ratios	95% C.I.	Odds Ratios	95% C.I.
Sex	2.0	0.7–6.0	1.9	0.6–6.2
Age	1.6 ***	1.1–2.4	1.6 ***	1.1–2.4
Ethnic cultural identity score	1.0	0.8–1.3	1.0	0.8–1.3
US, Mainland cultural identity score	1.0	0.8–1.2	0.9	0.8–1.2
“Extended family” household ^a^	0.6	0.2–2.0	0.6	0.2–4.7
“Parent(s) only or sibling(s) included” household ^b^				

*** indicates *p*-value < 0.05. ^a^ Households include members who are not parents or siblings. ^b^ reference group. Households that have parent(s) or parent(s) and sibling(s).

## Data Availability

The data presented in this study are available on request from the corresponding author. The data are not publicly available due to restrictions (e.g., privacy or ethical).
